# Agro-Environmental Determinants of Leptospirosis: A Retrospective Spatiotemporal Analysis (2004–2014) in Mahasarakham Province (Thailand)

**DOI:** 10.3390/tropicalmed6030115

**Published:** 2021-06-28

**Authors:** Jaruwan Viroj, Julien Claude, Claire Lajaunie, Julien Cappelle, Anamika Kritiyakan, Pornsit Thuainan, Worachead Chewnarupai, Serge Morand

**Affiliations:** 1 Faculty of Public Health, Mahasarakham University, Mahasarakham 44150, Thailand; jaruwan.v@msu.ac.th; 2 Institut des Sciences de l’Evolution, CNRS/UM/IRD/EPHE, Montpellier Université, 35095 Montpellier, France; julien.claude@umontpellier.fr; 3 Inserm, UMR LPED (IRD, Aix-Marseille Université), 13001 Marseille, France; claire.lajaunie@inserm.fr; 4 CIRAD, UMR ASTRE, 34398 Montpellier, France; julien.cappelle@cirad.fr; 5 UMR EpiA, INRA, VetAgro Sup, 69280 Marcy l’Etoile, France; 6 Faculty of Veterinary Technology, Kasetsart University, Bangkok 10200, Thailand; anamika.k@ku.ac.th; 7 Mahasarakham Provincial Public Health Office, Mahasarakham 44000, Thailand; pornanun@gmail.com; 8 Ban Ke Health Promotion Hospital, Mahasarakham 44150, Thailand; chead_13@hotmail.com

**Keywords:** leptospirosis, public health, One Health, livestock, spatiotemporal analysis, general additive modeling, Thailand

## Abstract

Leptospirosis has been recognized as a major public health concern in Thailand following dramatic outbreaks. We analyzed human leptospirosis incidence between 2004 and 2014 in Mahasarakham province, Northeastern Thailand, in order to identify the agronomical and environmental factors likely to explain incidence at the level of 133 sub-districts and 1982 villages of the province. We performed general additive modeling (GAM) in order to take the spatial-temporal epidemiological dynamics into account. The results of GAM analyses showed that the average slope, population size, pig density, cow density and flood cover were significantly associated with leptospirosis occurrence in a district. Our results stress the importance of livestock favoring leptospirosis transmission to humans and suggest that prevention and control of leptospirosis need strong intersectoral collaboration between the public health, the livestock department and local communities. More specifically, such collaboration should integrate leptospirosis surveillance in both public and animal health for a better control of diseases in livestock while promoting public health prevention as encouraged by the One Health approach.

## 1. Introduction

Human leptospirosis is a neglected infectious disease [1] with high incidence in 34 countries [2]. High prevalence of human leptospirosis occurs in tropical environments where conditions may favor the survival of *Leptospira* in the environment [3,4]. Leptospirosis is a zoonotic bacterial disease caused by spirochete species of the genus *Leptospira* [5], which includes nine pathogenic species and at least five intermediate pathogenic ones [6]. The clinical manifestations in humans are broad such as febrile illness. However, some patients may develop icteric leptospirosis, which is characterized by a combination of hepatic and renal impairment, hemorrhage, and vascular collapse [7,8]. Exposure to virulent leptospires may be direct, via contact with urine or tissues from infected animals, or indirect, via water contaminated with leptospires shed by infected animal. Many animal species shed leptospires [6] with an important role as reservoir for livestock [9]. Wild rodents have usually been considered as one of the main reservoirs for human leptospirosis [10], although several studies have recently challenged their importance in rural environments compared to urban environments [11,12,13].

Leptospirosis incidence in Thailand shows a strong seasonality with a high incidence during the wet season [14]. Environmental factors, climate variability and extreme events resulting in flooding events may contribute to leptospirosis transmission and increased infection risk [15,16,17]. Leptospirosis outbreaks have often been related to heavy rainfall, which conditions may favor the survival and dispersion of *Leptospira* species in the environment.

In Asia, leptospirosis incidences range from 0.1 to 10 cases per 100,000 persons but can reach over 50 per 100,000 per year during outbreaks [18], although the burden of leptospirosis differs from country to country depending on the quality of the disease surveillance and health prevention. In Asia, leptospirosis incidence has been related both to the monsoon and extreme events [14,15], exposure to moist soils [19] and poor sanitary conditions [20].

In Thailand, the average annual incidence rate was around 6.6 cases per 100,000 persons during from 2003 to 2012 [21]. Leptospirosis was identified a major problem as early as 1943, following a big flood event that hit Bangkok. In 1972, leptospirosis was included as one of the 58 reportable infectious diseases under the National Passive Surveillance System [22]. Leptospirosis re-emerged in the mid- 1990s with a major outbreak occurring in 2000. In 2012 a big flood event hit Bangkok, but a retrospective study concluded to a lack of statistical association between the locations of human leptospirosis cases and the flooded areas [23]. 

The first report in Mahasarakham province (Northeastern Thailand) in 1996 reported 12 patients. In 1997, the number of diagnostic cases increased up to 300 [24]. Leptospirosis is considered as endemic and ranked as a significant public health concern in this rural province of Thailand [25], with most citizens living from agriculture (43.92%) [26,27]. In that province, the Provincial Public Health Office, the Livestock Department, the Agriculture Department has tried to control this disease by employing people to eradicate rats in infected areas, by providing health education, by establishing a surveillance system and by setting the Surveillance and Rapid Response Team or SRRT in every district of the province under the District Strengthening Disease Control Program. Because of the still occurring transmission of leptospirosis, the province has implemented new objectives to reduce the incidence of the disease by improving the surveillance and reporting, taking environmental and lifestyle changes into account, and providing health education through community engagement. Until the year 2010, the province Mahasarakaham has followed the guideline of the Minister of Public health of Thailand which focused on rapid disease control with “war room” and Special Response Team established to handle leptospirosis outbreaks. In 2011, the province has modified its prevention strategy by increasing the participation of Public Health Department, the Livestock Department, the Agriculture Department and the Tambon Administration Organization [28].

The aim of the present study is to investigate the factors, including human population size, livestock, rainfall, flood cover and physical geography, i.e. average slope, that can explain the spatiotemporal distribution of human leptospirosis cases in Mahasarakham, Thailand from 2004 to 2014. We conducted our analyses following the following workflow: first (1), we investigated the spatial autocorrelation of leptospirosis cases and performed spatial interpolation; second (2), we analyzed the temporal pattern of leptospirosis cases; third (3), we described the spatiotemporal distribution of cases and tested for correlation between leptospirosis cases, rainfall and flood cover; fourth (4), we tested the effect of health policy and surveillance system change in 2012 on the temporal dynamics; and finally (5), we used general additive modeling (GAM) to investigate the effects of rainfall, flood cover and livestock on leptospirosis cases taking into account the results obtained from (1), (2), (3) and (4). 

## 2. Materials and Methods

### 2.1. Study Location

**Figure 1:**
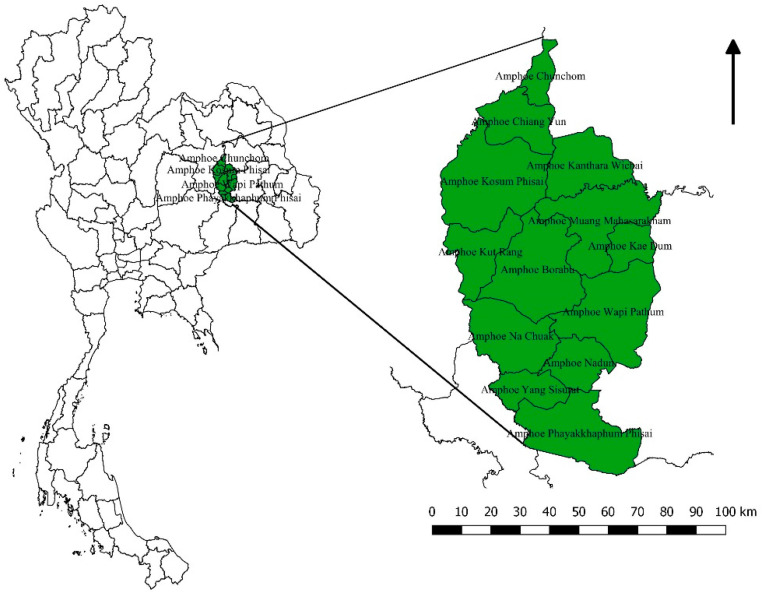
Location of the province in Thailand (in green).

**Figure 2:**
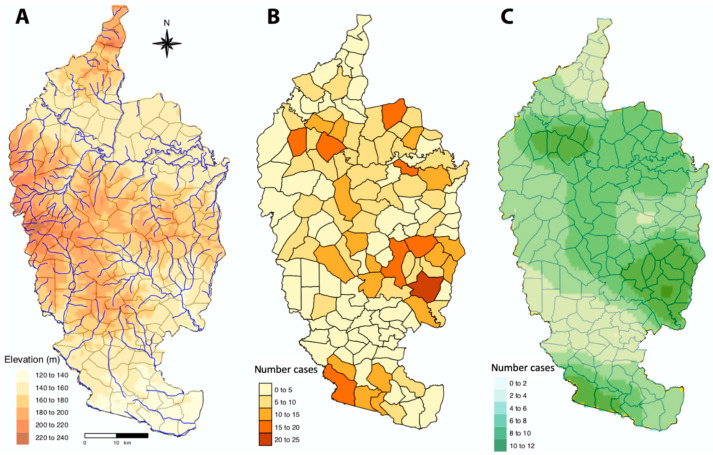
Mahasarakham province with: (**A**) geography with elevation, main rivers and sub-district boundaries; (**B**) overall leptospirosis cases over 2004–2014 period per sub-district (with localisation of Wapi Pathum district, Kosum Phisai district and Chuen Chom district, cited in the results section) (**C**) interpolation of leptospirosis cases at the level of subdistrict by kriging using semi-variogram based on the centroids of geographical coordinates of each sub-district.

Mahasarakham province is located in the Northeast of Thailand located in the middle of the Khorat plateau (Figure 1). The climate is of savannah type with a rainy season spanning from May to November. There are 3 main rivers: Chee, Seaw, and Choo all tributes of the Mekong River [25]. This province covers 5300 square km lying within the 15°25–16°40 N and 102°50–103°30 E with low elevation variation (130 to 230 meters) (Figure 2A). The province is divided into 13 districts, 133 sub-districts comprising 1982 villages. Last census of 2016 gave a total population of 869,280 persons and 244,155 households in 2016 [26].

### 2.2. Leptospirosis Prevention and Control Implementation

From 2004 to 2010 the province followed the guideline of the Minister of Public health of Thailand, a Special Response Team established to handle leptospirosis outbreaks. In this period, the public health department was the main department involved in leptospirosis prevention and control, working jointly with livestock department only, to investigate in the case of an outbreak. Starting in 2011, the province modified its prevention strategy by increasing the participation of the Public Health Department, the Livestock Department, the Agriculture Department and the Tambon Administration Organization. The main actions were dedicated to prevention education (wearing of boots in rice fields) and rodent control by the villagers. They have been involved in establishing leptospirosis prevention and control plan, and developed activities such as improved reporting in a One Health approach, rodent control or health education [28]. 

### 2.3. Data Used in the Study

#### 2.3.1. Human Leptospirosis Cases

Data on leptospirosis cases were obtained for each of the 133 sub-districts and the 1982 villages, for the years 2004–2014 from Public Health Department of Mahasarakham province, Thailand (Figure 2B). This research includes all cases of leptospirosis obtained from the Department of Public Health of Mahasarakham Province. The cases of leptospirosis have been clinically diagnosed in hospitals by trained clinicians: patients used to work in paddy, damp and swampy places, presented signs of headaches, high fever and muscle pain. Only patients with severe symptoms have been laboratory confirmed for leptospirosis. Leptospirosis cases are then symptomatic cases obtained through passive surveillance and may not reflect the true incidence due to underreporting of asymptomatic cases.

#### 2.3.2. Land Use and Land Cover

Land use was obtained from the Minister of Natural Resources and Environment, Thailand [29] (rivers, administrative boundaries, average slope).

#### 2.3.3. Seasonality and Rainfall

The climate of Mahasarakham is impacted by the monsoon and can be divided in 3 seasons: the summer season occurs from February to May, the wet season from May to October and the winter from November to January. Data on monthly rainfall data were obtained from the Thai Meteorological Department, Ministry of Information and Communication Technology.

#### 2.3.4. Flood Cover

Flooding information from 2004–2014 was obtained using satellite remote sensing. We used MODIS TERRA MOD09A1 Surface-Reflectance Product, with a spatial resolution of 500m and a temporal resolution of 10 days, from the Land Processes Distributed Active Archive Center of NASA (National Aeronautics and Space Administration). We used the Modified Normalized Difference Vegetation Index (MNDVI) to determine the flooded status of a 500 m pixel as validated and used in previous works focusing on flood-driven leptospirosis in Cambodia [30] and Thailand [31]. The flood cover was estimated at the sub-district level as the proportion of 500 m pixels with the flooded status in the sub-district.

#### 2.3.5. Livestock Data

**Figure 3:**
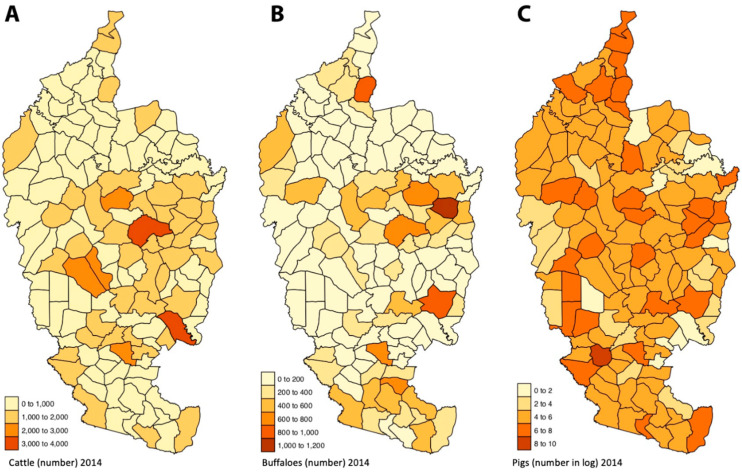
Mahasarakham province with (**A**) number of cattle, (**B**) number of buffaloes, (**C**) number pigs (in log due to the over-dispersion of the number of pigs among districts) per district in 2014.

The number of cattle, buffaloes, and pigs per sub-district were obtained from the Livestock Department of Mahasarakham for the year 2014 (Figure 3), respectively 121,841 heads of cattle in total, 26,602 buffaloes and 52,617 pigs in total.

#### 2.3.6. Data Integration

Data were obtained at different levels: leptospirosis cases at village level, livestock number at sub-district level, population size at sub-district level for the year 2014, the average slope at the sub-district level, flood cover percentage of the sub-district (see Section 2.3.3), rainfall (see Section 2.3.2) at provincial level. We analyzed the data at the sub-district level. For that, we aggregated the leptospirosis cases at the sub-district level by summing cases of all villages of the sub-district and we considered similar rainfall values for each sub-district.

### 2.4. Statistical Analyses

All analyses were conducted using the freeware R [32].

#### 2.4.1. Spatial Autocorrelation of Leptospirosis Cases

Correlogram analysis was used to identify spatial autocorrelation. Moran’s I test was performed to test the significance of the correlations with spatialEco implemented in R [33]. We also studied the autocorrelation by using sub-district as units and using the lag in terms of neighboring with the R spData package [34]. We performed the analysis for the period 2004–2014 and also for the three years showing the highest number of cases.

#### 2.4.2. Semi-Variogram and Kriging Interpolation of Leptospirosis Cases

Kriging involves including a fixed number of nearest neighbor points within a fixed radius [35] and relies on semi-variograms that quantify spatial autocorrelation among all pairs of data according to distance [36]. Semi-variograms were estimated from survey data by calculating the squared difference of leptospirosis cases between all pairs of subdistrict centroid. Semi-variance values were grouped and averaged according to separation distance (lags). Semi-variogram model and kriging were obtained using the packages FRK and INLA implemented in R [37,38].

#### 2.4.3. Temporal Analysis and Temporal Auto-Correlation

We used time-series analysis to study the patterns of leptospirosis cases during the study period. Exponential smoothing model was used to assess temporal trends in the overall rates of leptospirosis cases using ncf implemented in R [39]. The time series included 132 months in total from January 2004 to December 2014. To determine the general form of the model to be fitted, residual ACF (Autocorrelation Function) was examined. Considering the ACF graphs, different ARIMA models were identified for model selection. The series were then decomposed with a moving average taking into account a period of one year using the package stats, function decompose, implemented in R [40].

Similarly, we analyzed the temporal variations of rainfall (see Section 2.3.3*)* and flood cover (see Section 2.3.4*)* (as mean percentage computed over sub-districts) using the same methodology as above. The function ccf was used to compute the cross-correlation or cross-covariance between univariate series, i.e., rainfall and leptospirosis incidence, rainfall and flood cover and leptospirosis incidence.

We used wavelet analysis which transforms function to decompose a time series to reveal periodic signals at each time point in the series. The wavelet analysis coefficients showed magnitudes of correlation of the leptospirosis incidence for each year and period length and were displayed using a power spectrum over the full time series. We used the packages biwavelet and WaveletComp [41,42] implemented in R.

#### 2.4.4. Association between Rainfall, Flood Cover and Leptospirosis Cases 

We used the above time-series analysis to study the patterns of leptospirosis cases during the study period using ACF with investigated correlation lag and the correlation values at the bet lag period (in month) among rainfall, flood cover and leptospirosis cases.

#### 2.4.5. Causality of Public Health Change on the Temporal Dynamics

We investigated the causal effect of a designed intervention on a time series using the package CausalImpact implemented in R [42]. Given a response time series, the method implemented in CausalImpact constructs a Bayesian structural time-series model that predicts how a response metric would have evolved after an intervention and if this intervention had never occurred [43]. 

#### 2.4.6. Spatial Analysis

Factors that could likely be in association with human leptospirosis cases including pig number, cattle number, buffalo number, population size, were mapped at the sub-district level. The rgdal and tmap packages [44,45] implemented in R were used.

#### 2.4.7. Association between Leptospirosis Cases and Investigated Factors Using General Additive Modeling

General Additive Modeling (GAM) is an extension of the generalized linear models with the adaptability for non-normally distributed variables. The model assumes that the response variable, here leptospirosis cases per sub-district and per month, is dependent on the univariate smooth-terms of independent variables [46]: population size, number of cattle, number of pigs, number of buffaloes, rainfall, average slope and percentage of flooded area per sub-district. Investigating the number of cases allowed to investigate the smooth effect of population size, e.g., the transition from rural to urban population. All models were fitted using the MGCV package implemented in R [47]. We used the function gam.check to choose the basis dimension for each predictor according to estimated degrees of freedom value in the main effect. Outputs of GAM models were obtained using the package gratia implemented in R [48].

## 3. Results

### 3.1. Human Leptospirosis Incidence

The total number of leptospirosis human cases recorded from 2004–2014 was 762, with the highest number (177) recorded in 2012 followed by year 2009 (90) and 2014 (79). The annual incidence rate was 7.97 cases per 100,000 and ranged from 3.80 to 20.36 cases per 100,000. Non-significant difference (*p* = 0.09) was detected in comparison to the average annual leptospirosis incidence rate of Thailand, which was 6.6 per 100,000 population [21].

The largest number of cases were found in Wapi Pathum district (160), followed by Kosum Phisai district (123) which are all located in low elevation. Chuen Chom district (North of the province) had the lowest number of cases over the considered period (3) and is located at higher elevation (Figure 2B). 

Two areas showed nearly no cases, they corresponded to Chuen Chom district in the north and to the Eastern part of Na Chuak district; places with the largest incidences were situated in the east (Wapi Pathum district, Kea Dum district), in the south (Phayakkhaphum Phisai district), in the west (Borabu district) and in the east of Kosum Phisai district.

### 3.2. Spatial Autocorrelation of Leptospirosis Cases

Pooling human leptospirosis cases for the 2004–2014 period revealed a weak local spatial autocorrelation, not significant after 20 km (see Supplementary Materials). Local autocorrelation nerveless increased during two years of highest incidence (2012 and 2014), suggesting the existence of a pattern of spatial variation changing through time. During these two years, autocorrelation was significant until spatial lag ranging from 20 to 40 km (size of two adjacent sub-districts). This observation was not verified in 2009, a year characterized by its high incidence (see Supplementary Materials). When analyzing data considering the order of neighborhood between sub-district, a positive autocorrelation was found at the first and second order while considering data during the whole-time study. In 2012 during highest number of cases, a similar but stronger pattern was found at the first and second order also. In 2009 during the second highest number of cases, such autocorrelation was not observed. However, in 2014 during the third highest number of cases, a similar pattern was found at the first and second order (see Supplementary Materials).

### 3.3. Semi-Variogram and Kriging of Incidence

The spatial distribution of the human incidence among sub-districts (Figure 2B) was analyzed using semi-variogram analysis with the best model function spherical. The kriging interpolation, using the results of the semi-variogram analysis, is represented in Figure 2C. A high spatialized interpolation of leptospirosis incidence corresponds to low elevation area (Figure 2A).

### 3.4. Time Series Analysis of Leptospirosis Cases

**Figure 4:**
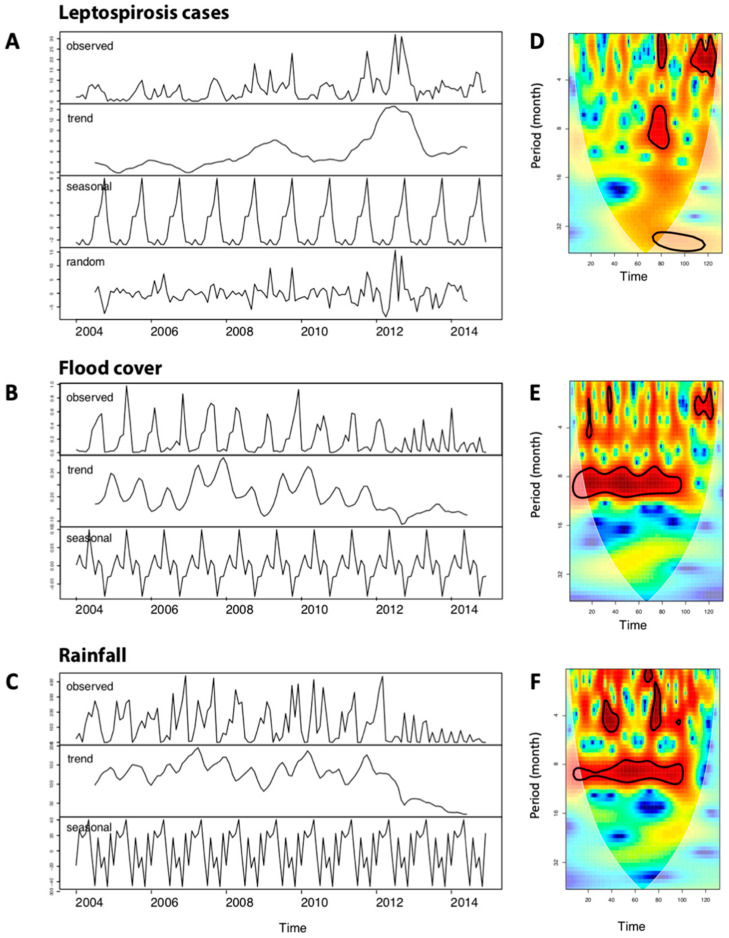
Temporal analysis over the period 2004–2014 of: (**A**) observed leptospirosis cases by month decomposed in smooth trend, seasonal and random effects; (**B**) flood cover decomposed in smooth trend and seasonal effect; (**C**) rainfall decomposed in smooth trend and seasonal effect. Wavelet power spectrum of (**A**) observed leptospirosis cases, (**B**) rainfall, (**C**) flood cover over 2004–2014 (132 months). Wavelet power values increased from blue to red, and black contour lines indicate the 5% significance level. In this example, the time-series show a significant 12 month periodicity over 2009–2010 observed leptospirosis cases (**D**) and over for 2004–2012 for flood-cover (**E**) and rainfall (**F**).

There was an increasing trend in leptospirosis cases from 2004 to 2012 (Figure 4A) with a sharp decrease at the end of 2012, followed by a slight increase in 2013 and 2014. There was a strong seasonal pattern with a large number of leptospirosis cases during the rainy season (June to October) but decreased in winter until the end of the dry season (Figure 4A). The ACF graph showed seasonality in leptospirosis cases. However, the wavelet analysis (Figure 4D) did not confirm a seasonal pattern at the exception of years 2009–2010.

### 3.5. Time Series Analysis of Rainfall and Flood Cover

Using a similar method as above, we found a strong seasonal pattern of rainfall and flood cover (Figure 4E,F). A strong decrease in both rainfall and flood cover occurred after 2012, suggesting that Mahasarakham was entering into a drought period. Wavelet power spectrum (Figure 4E,F) confirmed the above observation by revealing significant 12-month periodicity over the entire time period. However, the seasonal autocorrelation disappeared from 2012 to 2014 for both rainfall (Figure 4E) and flood cover (Figure 4F), confirming the time series trends (Figure 4B,C).

### 3.6. Cross Temporal Correlation Analysis

**Figure 5:**
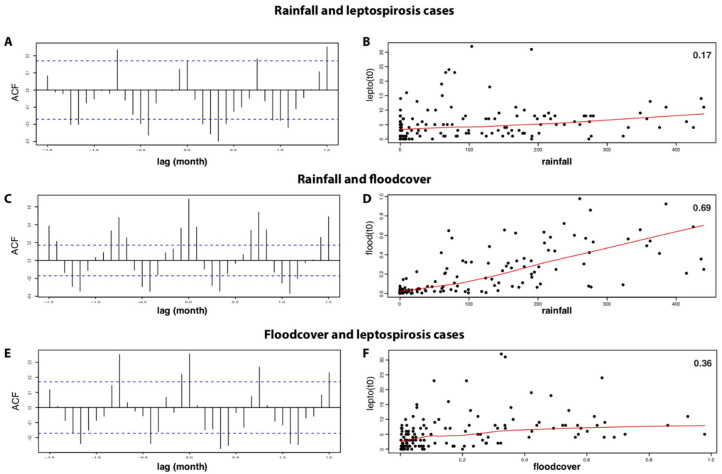
Temporal correlation over the period 2004–2014 between: (**A**,**B**) rainfall and leptospirosis cases with lag <1 month (**A**) and R= 0.17 (**B**); (**C**,**D**) rainfall and flood cover with lag <1 month (**C**) and R= 0.69 (**D**); (**E**,**F**) flood cover and leptospirosis cases with lag <1 month (**E**) and R= 0.36 (**F**).

Cross-correlation analysis among pairs of univariate series, leptospirosis cases, rainfall and flood cover, revealed a lag time of less than one month (Figure 5). Significant correlation among pairs of univariate series was observed with higher correlation between rainfall and flood cover (R = 0.69) than between rainfall and leptospirosis cases (R = 0.17). 

### 3.7. Impact of the Surveillance System

**Figure 6:**
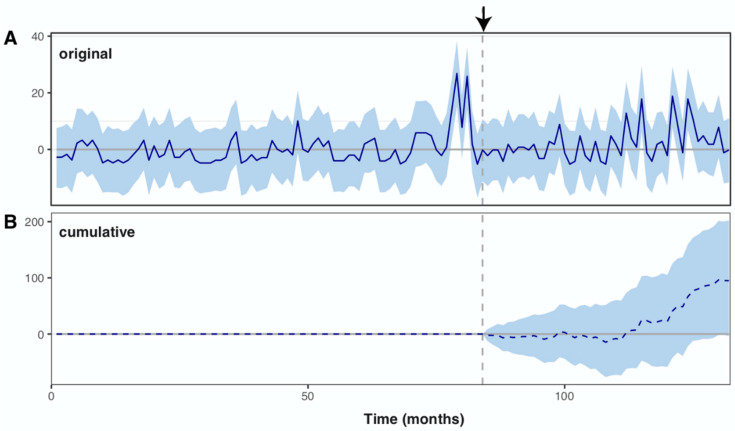
Causal effect of the change in health surveillance system after 2010 (**A**. Original, **B**. Cumulative). Significant causal effect of the change in public health policies was assessed using Bayesian one-sided tail-area probability (*p* = 0.03). The relative number of leptospirosis cases showed an increase of 38%, however, the 95% credible interval on this increase overlapped the 0 [−1%, +81%] during the whole time observed after the change in surveillance system (the arrow on the top of the dashed line indicates the change in health surveillance system).

There was no significant decrease of incidence after 2011 when public health policies have changed by improving the health surveillance. Rather, an increase in the number of cases was observed (Figure 6A). A significant causal effect of the change in public health policies after the high incidence of 2010 (Figure 6B) was noted (Bayesian one-sided tail-area probability *p* = 0.03 with a posterior probability of a causal effect: 96.967%). In relative terms, the number of leptospirosis cases showed an increase of 38%, however with a large credible interval that was still crossing the 0 increase even when considering the entire post-intervention period after 2011 (i.e., 2012–2014). The increasing trend of leptospirosis cases reported in Figure 4A, while rainfall and flooding decreased (Figure 4B,C), is then explained by a better case reporting initiated in 2011.

### 3.8. Association between Leptospirosis Occurrence and Explanatory Factors Using General Additive Modeling

We developed the following initial GAM model that took into account the spatiotemporal dynamics of leptospirosis cases and the temporal dynamics of rainfall (see Section 2.3.4) and flood cover (see Section 2.3.4), and the potential explanatory variables using a negative binomial function (with theta estimated before) (Table 1). 

However, this model explained only 14% of the deviance (Table 1).

## 4. Discussion

We investigated the spatiotemporal distribution of leptospirosis in Mahasarakham province over 11 years and identified several factors associated with human leptospirosis incidence. Mahasarakham province, with an average annual leptospirosis incidence of 7.97 cases per 100,000 people did not differ significantly from the average annual leptospirosis incidence of Thailand. Nevertheless, Mahasarakham was chosen as a pilot province for the implementation of surveillance and control of leptospirosis [28] and the present study contributes to the assessment of the public health policy.

The highest number of leptospirosis cases was reported in Mahasarakham province in 2012, one year after Mahasarakham province has implemented a new leptospirosis prevention and control plan with the Surveillance and Rapid Response Team (SRRT) and the District Strengthening Disease Control. The evolution of the prevention and control of leptospirosis in the province of Mahasarakham is extensively exposed by Viroj et al. [49]. The prevention and control of leptospirosis followed the 4E2C procedure (Early detection; Early diagnosis; Early treatment; Early control; Coordination; Community involvement) established by the Ministry of public health [49]. A new plan implemented in 2011 aimed at increasing collaboration between the Public Health Department, the Livestock Department, the Department of Agriculture and the Tambon Administration Organization for better prevention and control leptospirosis [50]. Moreover, the new implementation also aimed at improving the quality of data collection and reporting on fatality rates and causes. Hence, the increase of the number of leptospirosis cases recorded in 2012 may partially be due to a better reporting in the province. In this study we found that the new leptospirosis prevention and control plan on leptospirosis was associated with an increase of incidence, independently of environmental factors (rainfall, flood cover, livestock), although the significance of this impact on middle term necessitates more years of observation. This unexpected effect may result from a better documentation of cases, since the new leptospirosis prevention and control plan has increased participation, awareness and better communication towards the health officers, the district hospitals and the local communities. We believe that, at least, the new plan could increase the number of cases reported.

### 4.1. Temporal Analysis of Leptospirosis

Leptospirosis cases dramatically increased in the wet season and had the highest rate at the end of the rainy season as showed by the time series analysis. This observation is similar with numerous studies showing that most leptospirosis cases occurred during the rainy season [14,51,52]. During the rainy season the soil is moist and allows the bacterial leptospires to survive longer outside while water flushes and flooding might help their dispersion [53]. The transmission dynamics between humans, animals, and a contaminated environment could be enhanced by flooding events [54]. In complement, the results of the time series analyses showed the likely association between rainfall, flood areas and leptospirosis cases, although flood cover explained better the number of leptospirosis cases than rainfall. 

### 4.2. Spatial Analyses of Leptospirosis

The low local spatial autocorrelation, inferior to 20 km, is also in agreement with other studies on environmentally transmitted diseases such as leptospirosis. This result is similar with a study undergone in Netherlands where autocorrelation of leptospirosis incidence was about 12 km [55]. Local autocorrelation nevertheless increased during the two years of highest incidence (2012 and 2009), suggesting that patterns of spatial variation exist. Positive autocorrelation was found at the first and second district neighborhood order while considering the whole time period showing that disease transmission commonly occurred among adjacent districts as also observed using kriging interpolation.

### 4.3. Likely Agro-Environmental Determinants of Leptospirosis

**Figure 7:**
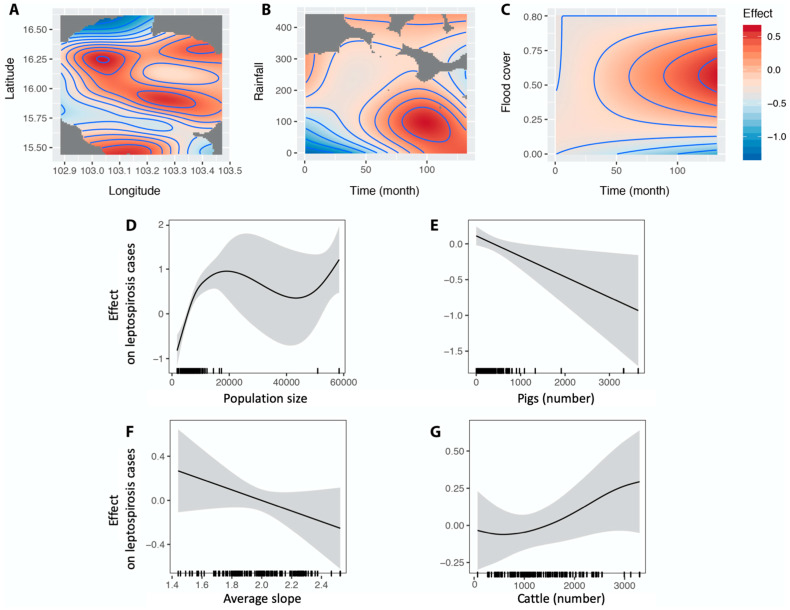
Results of the best General Additive Modeling explaining the number of cases of leptopsirosis in Mahasarakham province over 2004–2014 by sub-district and by month, using a binomial negative link function (with theta = 0.267) (see Table 1). The effect, explaining the number of cases, of smoothed variables selected in the best GAM were (**A**) the geographical distribution of subdistrict (given by longitude and latitude of the centroid), (**B**) rainfall controlling for time (month); (**C**) percentage of flooded areas controlling for time (month); (**D**) population size per sub-district; (**E**) pig number; (**F**) average slope of the sub-district; (**G**) cattle number.

GAM analysis allowed us to consider the spatiotemporal dynamics of the leptospirosis transmission revealed by the kriging and time series analyses. The best GAM model confirmed the significant influence of geography (matrix of geographic coordinates of sub-districts), rainfall, slope and flood cover, population size and number of cattle and pigs. The partial contribution of geography to the variable response (Table 1, Figure 7A), i.e., leptospirosis cases, reflected the kriging map (Figure 2C), while the greatest effects of the partial contributions of rainfall and of flooded cover (controlling for time) occurred in 2012 and later. 

Average slope of the district was negatively associated with human leptospirosis incidence. Flat topography allows the formation of permanent puddles, which is strongly associated with the formation of flooded areas favoring the transmission of leptospires through contaminated water [56,57]. However, some studies did not find a significant association between flooded areas and leptospirosis incidence such as in Thailand during the flooding event of 2012 [23]. Thaipadungpanit et al. [58] hypothesized that long and intense flooding may lead to a dilution of leptospires reducing the risk of transmission during this long flooding event. Indeed, the GAM model showed that the effect of the partial contributions of flood cover (controlling for time) decreased for both low and large flooded areas (Table 1, Figure 7C).

Population size was positively and significantly associated with leptospirosis cases. A pattern commonly found for environmentally transmitted diseases [59]. Human demography is an important driver of infectious diseases in Southeast Asia [60]. Moreover, population size and population growth are typically associated with agricultural intensification and increasing farming that may enhance leptospirosis transmission [61].

Our results showed a positive association between cattle number and leptospirosis cases. Cattle are well-known as carriers of leptospires [9,62] and a strong association between cattle number and leptospirosis transmission has already been reported [63]. Free-ranging cattle systems can increase transmission of leptospirosis by exposing human to contaminated environment by cattle urine and leptospires [55]. Water-source sharing of cattle and human can influence the risk of *Leptospira* exposure [64]. In low-income rural communities, cattle appear more important than rats for maintaining the transmission of leptospirosis [65]. Grazing range characteristics are likely to play a role in shedding the concentration of leptospires in the environment [66,67]. In Thailand, the dominant serovar Shermani observed in cattle was also observed in humans [9]. The importance of cattle as a determinant of leptospirosis occurrence in Mahasarakham finds its confirmation by the fact that farmers are the main people infected by leptospirosis. Most of raising cattle in Mahasarakham cattle were taken to the grassland in the village which increased spared *Leptospira* in water-source and environment. 

Buffaloes could play a role as an important leptospirosis animal reservoir in Thailand. The serovars found in buffaloes, such as Shermani, Pomona, Sejroe, Bratislava and Bataviae can be pathogenic in humans [9]. However, our results did not support a role for buffaloes in the transmission of the disease. 

Backyard piggeries with poor sewage management have been implicated in leptospirosis transmission [68]. However, our results showed a negative association between the density of pigs and the number of leptospirosis cases. As commonly observed in Northeast Thailand, most pig farming in Mahasarakham province consist of small backyard stables, in which pigs are supposed to have low exposure to environmental pathogens contrary to cattle. But the negative effect of pig number on the leptospirosis transmission could be explained by the intensive and misuse of antibiotics in backyard and small pig farming [69]. Our hypothesis is then that the release of antibiotics in the pig manure may contaminate the surrounding environment and may decrease the survival of environmental leptospires, ultimately leading to a decrease in their transmission. An ongoing study on the use of antibiotics in small and medium-sized pig and chicken farms in Nan province shows that several classes of antibiotics were widely used by local farmers [70]. These antibiotics are inexpensive and readily available.

The low explanatory power of the GAM stresses some missing factors in the understanding of the transmission dynamics of leptospirosis. The most important one is the human individual risk behavior which could not be integrated in the modeling framework used in this study, but nevertheless, human factors need to be considered in any strategic planning for disease prevention.

### 4.4. Implications for Leptospirosis Surveillance and Control

Leptospirosis incidence trend did not show any decrease in Mahasarakham province after the implementation of a new leptospirosis prevention and control plan. Leptospirosis prevention and control implementation requires strong strategies to improve surveillance, health education, and good practices to avoid sources of contamination [71,72]. The findings of our study give some evidence that environment, climate and livestock contribute to leptospirosis transmission to humans. This retrospective analysis may help at targeting sub-districts presenting environmental and farming characteristics, associated with a higher risk of leptospirosis transmission to humans, for improvement of leptospirosis prevention and control in Mahasarakham province, notably by using the One Health approach to improve multi-sectoral collaboration between public health, livestock department, department of agriculture, district hospitals and local communities in order to promote public and animal health educational activities. For this, the implementation must improve the control of leptospirosis infection at the interface of humans, animals and the environment by involving all stakeholders. Among measures, we can cite: vaccination of livestock, improvement of agricultural practices and safety, better access to drinking water, better wastewater management, an improved One Health surveillance system.

### 4.5. Strength and Limitations of the Study

This study investigates various factors such as human demography, livestock and the likely environmental factors to explain the spatiotemporal distribution of human leptospirosis cases. Our results suggest that the evolution of the prevention and control of leptospirosis implemented in the province of Mahasarakham, and extensively discussed in [49], may have gained some efficiency by significantly increasing the number of reported cases and then better following the real epidemiology of leptospirosis. However, several factors were not investigated in this study. The first one concerns the role of rodents as important reservoirs of *Leptospira spp.* in leptospirosis transmission. Studies showed that the seroprevalence of *Leptospira* in rodents was quite high in Thailand with the highest prevalence found in northeast Thailand (from 3.6% to 7%), which suggests the importance of rodents in leptospirosis transmission in this area [18,73]. Second, socioeconomic factors should be taken into account, such as the access to safe water or the wastewater management. A recent study emphasized the lack of access to safe water in Northeast Thailand [64]. These knowledge gaps could be filled by carrying out intensive screening for rodents in sites with high and low transmission, supplemented by a survey of socio-economic factors (professional behavior, access to drinking water and water management). Third, knowledge on the epidemiology of leptospirosis suggests that human behavior is an important factor to take into account to explain much of human infection. Including data on human behavior, which were lacking in this study, should improve the predictive quality of the model while providing avenues for better leptospirosis prevention. 

Finally, even if the passive surveillance system tends to underestimate the real burden of leptospirosis, the involvement of primary care units and volunteers from health villages in raising awareness among local communities allows rapid prevention from the onset of leptospirosis outbreak.

## 5. Conclusions

Leptospirosis prevention and control seemed to have gained from the enhanced collaboration between Public Health Department, the Livestock Department and the Tambon Administration Organization, through the new prevention strategy of 2011. The results of our study suggest targeting areas prone at risk, i.e., with high livestock or in flooded areas, complemented by improving communication to people at risk, i.e., farmers.

## Figures and Tables

**Table 1:** Results of the general additive modeling (GAM) explaining the number of cases of leptospirosis per subdistrict in Mahasarakham province using a negative binomial link (theta = 0.267), with approximate significance of smooth terms. Deviance explained = 14%, REML = 2950.7, AIC = 5852 (see Figure 7).

Terms	edf	Ref.df	Chi Square	*p* Value
S (longitude, latitude)	17.82	29	67.44	<0.0001
Te (month, rainfall)	12.28	24	94.43	<0.0001
Te (month, flood cover)	3.36	20	42.89	<0.0001
S (population)	3.98	9	72.42	<0.0001
S (pig number)	0.87	9	5.99	0.006
S (cow number)	1.26	9	3.44	0.037
S (average slope)	0.68	9	2.11	0.043

The best GAM model selected on the basis of AIC value included all initial variables except the number of buffaloes (Table 1, Figure 7): gam(leptospirosis cases per sub-district) ~ s(longitude, latitude) + te(month, rainfall) + te(month, flood cover) +s(number of people in sub-district)+s(number of pigs) + s( number of cattle) + s(average slope).

## Supplementary Materials

The following are available online at https://www.mdpi.com/article/10.3390/tropicalmed6030115/s1.
