# Supplementary material for: Agro-Environmental Determinants of Leptospirosis: A Retrospective Spatiotemporal Analysis (2004–2014) in Mahasarakham Province (Thailand)
Source: Trop Med Infect Dis. 2021 Jun 28;6(3):115. doi: 10.3390/tropicalmed6030115 (PMC8293432; doi:10.3390/tropicalmed6030115)
Supplement: Supplementary file 1 [file tropicalmed-06-00115-s001.zip › tropicalmed-1156964-supplementary.pdf]

Tropical Medicine and Infectious Disease

MDPI

Supplementary Materials

Spatial Autocorrelation of Leptospirosis Incidence

Correlograms were used to identify spatial autocorrelation with lag distance and the Moran's I test can be used to test the significance of the correlations. Spatial autocorrelation range was determined by checking at the significance value ($p = 0.05$) of the individual bins of lag distance at every 20 km.

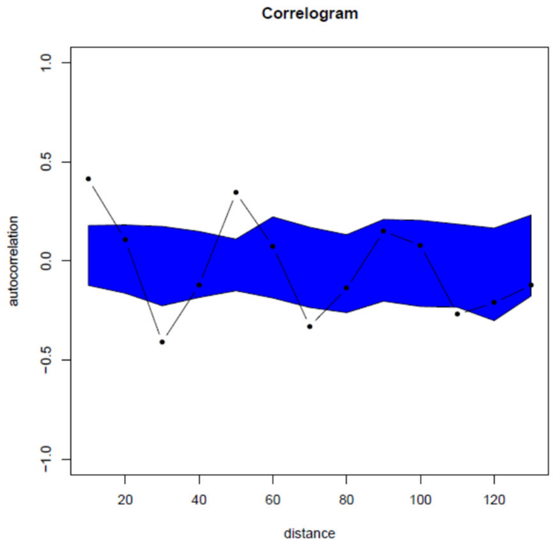

Auto correlation during 2004 – 2014

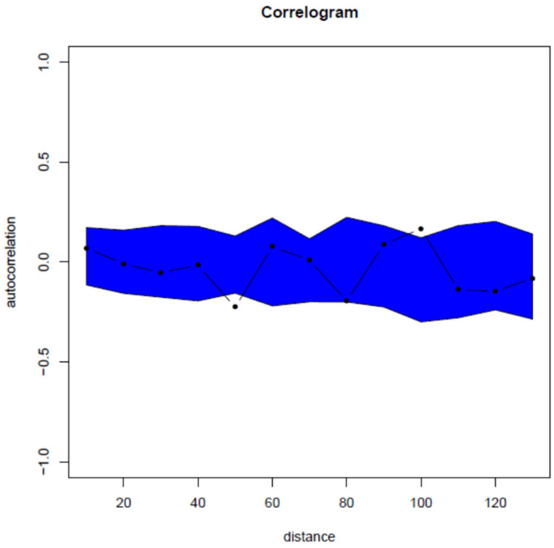

Auto correlation in 2009

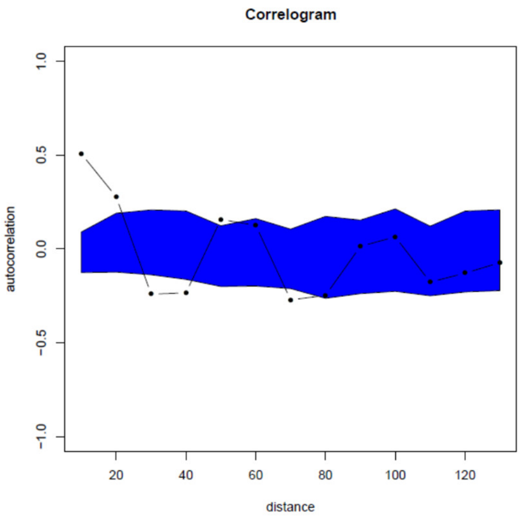

Auto correlation in 2012

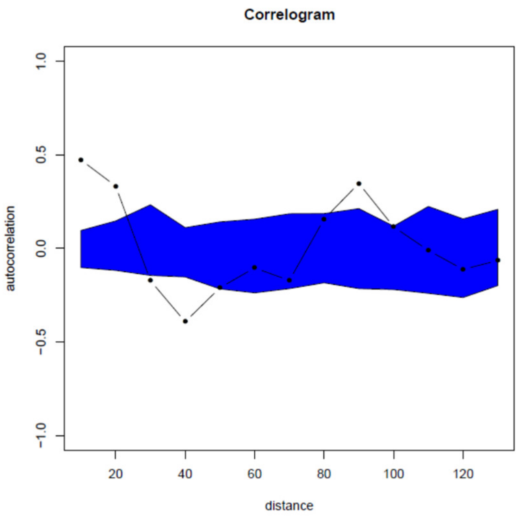

Auto correlation in 2014

Spatial autocorrelation analysis of neighbor of sub district showed autocorrelation in the first level ($p < 0.0001$) and the second level (P = 0.004) of sub district.

.Spatial autocorrelation of neighbor of sub district.
